# Expandable Constrained Transjugular Intrahepatic Portosystemic Shunt: An Individualised Approach for High‐Risk Patients?

**DOI:** 10.1111/liv.70436

**Published:** 2025-12-19

**Authors:** Hannah Rieland, Timo C. Meine, Anja Tiede, Jim B. Mauz, Heiner Wedemeyer, Jan B. Hinrichs, Frank K. Wacker, Benjamin Maasoumy

**Affiliations:** ^1^ Department of Gastroenterology, Hepatology, Infectious Diseases and Endocrinology Hannover Medical School Hannover Germany; ^2^ Institute for Diagnostic and Interventional Radiology Hannover Medical School Hannover Germany; ^3^ PRACTIS Clinician Scientist Program, Dean's Office for Academic Career Development Hannover Medical School Hannover Germany; ^4^ Department of Radiology St. Bernward Hospital Hildesheim Germany

**Keywords:** cardiac decompensation, contraindication, hepatic encephalopathy, portal hypertension, TIPS, transjugular intrahepatic portosystemic shunt

## Abstract

**Background and Aims:**

A transjugular intrahepatic portosystemic shunt (TIPS) can effectively overcome portal hypertension, but is prevented by contraindications in a considerable proportion of patients. In this study, we investigated the option of a constrained TIPS (cTIPS) with reduced diameter for high‐risk patients.

**Methods:**

Elective cTIPS were placed in 60 high‐risk patients at Hannover Medical School (10/2020–04/2023). In the cTIPS procedure, a 6 mm stent(−graft) was inserted first into the intrahepatic tract to constrain the TIPS endoprosthesis. Procedural data, complications and 12 months follow‐up were recorded. A control cohort of patients with standard TIPS (sTIPS) was generated using propensity score matching, resulting in 40 patients per group.

**Results:**

Indications for cTIPS were cardiac impairment, poor liver function and/or history of hepatic encephalopathy (HE) in 22, 17 and 8 patients, respectively. cTIPS was technically successful in all patients. Portosystemic gradient (PSG) was successfully lowered in both groups (sTIPS: median 60% reduction and cTIPS: median 50% reduction from an initial PSG of 15 mmHg in both groups, *p* = 0.006), while post‐TIPS PSG remained higher in the cTIPS group compared to the sTIPS group (cTIPS: median 7 mmHg vs. sTIPS: median 6 mmHg; *p* = 0.042). TIPS dysfunction and revision were significantly more frequent in the cTIPS cohort than in the sTIPS cohort (sTIPS: 1 vs. cTIPS: 9; *p* = 0.014 and sTIPS: 7 vs. cTIPS: 13; *p* = 0.036). Control of portal hypertensive symptoms (no paracentesis, no bleeding) was similar in both groups (*p* = 0.267), but overt HE was less frequent in the cTIPS group than in the sTIPS group (sTIPS: 14 vs. cTIPS: 4; *p* = 0.042).

**Conclusion:**

The constrained TIPS may offer a more careful step‐up approach for selected high‐risk TIPS patients. The technical procedure appears to be safe and feasible, however the risk for thrombosis and therefore needed revisions is increased. Larger study cohorts are needed to further explore promising results.

## Summary

- Transjugular intrahepatic portosystemic shunt (TIPS) placement is an effective treatment for portal hypertension, but poses the risk of clinical complications such as hepatic encephalopathy and cardiac or hepatic decompensation.
- Development of these clinical complications is correlated with the shunt volume and dependent on the diameter of the TIPS endoprosthesis, which is currently only available in diameters of 8 mm to 10 mm.
- In this study, we investigated a constrained TIPS technique with a smaller and size‐adjustable diameter of at least 6 mm in patients at high risk for clinical complications that may offer a more careful step‐up approach and could broaden the spectrum of patients eligible for TIPS.

**Abbreviations**
CD: cardiac decompansation
cTIPS: constrained transjugular intrahepatic portosystemic shunt
DAP: dose area product
DSA: digital subtraction angiography
HE: hepatic encephalopathy
PSG: portosystemic gradient
sTIPS: standard transjugular intrahepatic portosystemic shunt
TIPS: transjugular intrahepatic portosystemic shunt
VE: variceal embolization

## 1 Introduction

Transjugular intrahepatic portosystemic shunt (TIPS) placement is a highly effective treatment for variceal bleeding and refractory ascites in patients with liver cirrhosis and portal hypertension [1, 2, 3]. However, in a considerable proportion of the respective patients TIPS insertion is prevented by relative or absolute contraindications such as cardiac impairment, recurrent hepatic encephalopathy (HE) or advanced hepatic impairment. Nevertheless, TIPS often poses as the last treatment option besides liver transplantation [3].

Severe complications of TIPS placement such as HE and cardiac or hepatic decompensation have previously strongly correlated with the shunt volume and are therefore directly dependent on the diameter of the inserted TIPS [4, 5, 6, 7]. A recent study comparing 8 mm and 10 mm TIPS endoprosthesis has shown a similar shunt function, but a reduced rate of post TIPS HE in the 8 mm group [5]. Nevertheless, even an 8 mm portosystemic shunt might lead to a deterioration of liver or cardiac function and might worsen HE—especially in patients with relative contraindications for TIPS [8, 9, 10, 11, 12, 13]. Consequently, endovascular shunt reduction or occlusion may be needed at a later time [8, 9, 10, 11, 12, 13, 14, 15, 16].

Multiple shunt reduction techniques are described, including a parallel technique using a stent and a stent‐graft within the original TIPS, hourglass‐shaped stent‐grafts, a lasso technique and a sheath control technique also using stent‐grafts [10, 17, 18, 19]. These techniques can be cumbersome and challenging. Thus, the primary use of smaller size‐adjustable TIPS diameters with lower shunt volumes seems favorable. As a proposed solution, under‐dilatation of a commercially available 8 or 10 mm stent‐graft to only 6 mm has been described. However, this technique displayed uncontrolled passive expansion and is not reliable in achieving lower shunt volumes [5, 6, 20]. Of note, passive expansion was reported for both 10 mm Viatorr TIPS stent‐grafts underdilated to 8 mm and also novel controlled expansion Viatorr TIPS stent‐grafts underdilated to 6 mm diameter [21, 22]. In detail, passive expansion of controlled expansion Viatorr TIPS stent‐grafts underdilated to 6 mm diameter was reported with a median diameter of 7.9 mm at a median follow‐up of 171 days in the current study by Fonseca et al. [22]. In consideration of the early mortality within 3 to 12 months after TIPS placement in elderly patients reported by Saad et al. [23], passive expansion of controlled expansion Viatorr TIPS stent‐grafts underdilated to 6 mm diameter within 5–6 months could be a relevant problem. Therefore, constrained TIPS (cTIPS) techniques deploying a TIPS stent‐graft inside a 6 mm balloon expandable stent or stent‐graft, which have been reported in small case series [24, 25], might prevent passive expansion. This constrained technique might be beneficial in patients at risk for HE and/or cardiac decompensation (CD) and/or deterioration of liver function (DLF). However, more data are needed.

Hence, the purpose of our study has been to investigate the technical feasibility, procedural characteristics and clinical outcome of a TIPS constrained primarily to 6 mm with optional response‐guided controlled expansion in otherwise ineligible or high‐risk patients with portal hypertension compared to standard TIPS (sTIPS).

## 2 Materials and Methods

### 2.1 Data Collection and Study Population

Between 10/2020 and 04/2023, 60 patients with increased risk for adverse events underwent elective cTIPS placement with a diameter reduced to under 8 mm.

Data were obtained from radiological imaging or medical discharge and transfer reports, as well as from structured follow‐up in our outpatient clinic at 1, 3, 6 and 12 months after TIPS insertion [26, 27].

**FIGURE 1:**
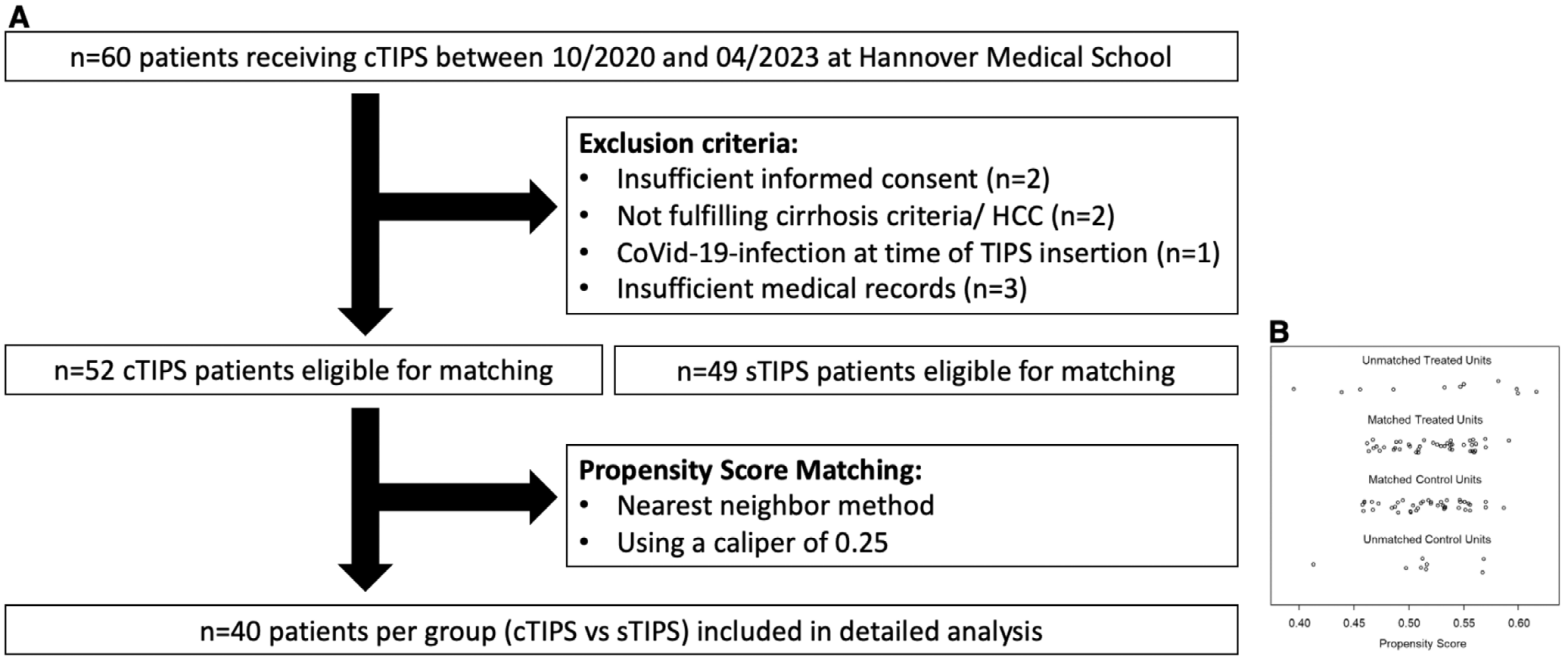
Selection algorithm for included constrained TIPS patients and generation of propensity matched standard TIPS cohort (A) Selection algorithm for cTIPS and sTIPS patients who were included in the analysis. (B) Distribution of propensity scores after applying nearest neighbour matching (caliper = 0.25) to the cTIPS (treated units) and sTIPS (control units) groups.

Inclusion criteria for this study were patients with ≥ 18 years of age, written informed consent and liver cirrhosis with portal hypertension. TIPS placement with an initial diameter of 6 mm was recommended by consensus of an interdisciplinary TIPS board (hepatology, radiology, transplantation) due to the patients being at increased risk for HE, CD or DLF in case of conventional TIPS placement. All of these patients had at least one relevant risk factor for the development of TIPS‐associated complications such as a history of overt HE, cardiac impairment and DLF (Bilirubin levels > 40 μmol/L and/or Cholinesterase < 2.5 kU/l) (Table 1A) [3, 26, 27, 28]. Exclusion criteria were absolute contraindications for TIPS according to EASL and German guidelines, insufficient informed consent, not fulfilling cirrhosis criteria, Covid‐19 infection, or HCC at the time of TIPS insertion, as well as insufficient medical records [3, 28] (Figure 1A).

**TABLE 1:** Demographic characteristics of patients treated with either standard TIPS or constrained TIPS at baseline.

	Standard TIPS(*n* = 40)	Constrained TIPS (*n* = 40)	*p*‐value
Age (years)	57.5 (47.0, 63.3)	60.5 (54.8, 67.3)	0.067
Sex (female/male) (*n*, %)	9/31 (22.5/77.5)	14/26 (35.0/65.0)	0.323
Aetiology of Cirrhosis			
ALD (*n*, %)	23 (57.5)	28 (70.0)	0.352
MetALD (*n*, %)	4 (10.0)	2 (5.0)	0.671
MASLD (*n*, %)	7 (17.5)	5 (12.5)	0.754
Viral (*n*, %)	4 (10.0)	5 (12.5)	1.000
Other (*n*, %)	8 (20.0)	8 (20.0)	1.000
TIPS Indication			
Refractory ascites (*n*, %)	32 (80.0)	32 (80.0)	1.000
Variceal bleeding (*n*, %)	15 (37.5)	10 (25.0)	0.335
Other (*n*, %)	1 (2.5)	4 (10.0)	0.356
Indication for cTIPS			
Cardiac impairment (*n*, %)		22 (55.0)	
Deterioration of Liver Function (*n*, %)		17 (42.5)	
HE (*n*, %)		8 (20.0)	
Baseline Characteristics			
PSE Test Score	−5.00 (−6.75, −1.25)	−6.00 (−10.00, −3.00)	0.047
Animal Naming Test	25.00 (21.50, 30.50)	21.00 (15.25, 23.00)	**0.010**
HE at baseline (*n*, %)	2/38 (5.0/95.0)	5/35 (12.5/87.5)	0.429
Previuos HE (*n*, %)	9/31 (22.5/77.5)	17/23 (42.5/57.5)	0.095
Previous variceal bleeding (*n*, %)	15/25 (37.5/62.5)	11/29 (27.5/72.5)	0.474
Ascites amount			**0.011**
None (*n*, %)	1 (2.5)	6 (15.0)	
Small (*n*, %)	8 (20.0)	1 (2.5)	
Medium (*n*, %)	4 (10.0)	9 (22.5)	
Large (*n*, %)	27 (67.5)	24 (60.0)	
MELD score	13 (9, 14)	12 (9, 15)	0.904
FIPS	−0.20 (−0.73, 0.33)	−0.05 (−0.75, 0.51)	0.501
Child Pugh Score	8 (7, 9)	8 (8, 9)	0.714
Child Class			0.696
A (*n*, %)	2 (5.0)	2 (5.0)	
B (*n*, %)	36 (90.0)	34 (85.0)	
C (*n*, %)	2 (5.0)	4 (10.0)	
Laboratory values			
Bilirubin (μmol/L)	16.5 (12.0, 22.3)	16.0 (9.8, 24.5)	0.821
Creatinine (μmol/L)	104.0 (76.8, 147.0)	108.5 (84.0, 144.0)	0.870
INR	1.21 (1.13, 1.35)	1.16 (1.11, 1.27)	0.252
Haemoglobin (g/dl)	9.7 (8.2, 11.6)	9.3 (8.3, 11.4)	0.658
Thrombocytes (10^3^/μL)	120 (75, 196)	112 (72, 198)	0.620
Leukocytes (10^3^/μL)	5.20 (3.68, 8.30)	5.55 (3.75, 7.62)	0.751
Sodium (mmol/L)	137 (133, 139)	134 (131, 138)	0.070
Albumin (g/L)	29.0 (26.8, 35.5)	31.0 (26.8, 35.3)	0.725
Cholinesterase (kU/L)	3.50 (2.39, 4.44)	2.17 (1.59, 3.11)	**0.005**
AST (U/L)	39 (31, 53)	37 (25, 48)	0.209
ALT (U/L)	23 (17, 33)	23 (14, 28)	0.631
AP (U/L)	130 (86, 165)	130 (99, 175)	0.542
γGT (U/L)	115 (54, 200)	128 (47, 234)	0.631
eGFR (ml/min)	68 (43, 99)	62 (41, 81)	0.502
NT‐proBNP (ng/L)	131 (91, 280)	202 (120, 383)	0.435
CRP (mg/L)	10.4 (3.4, 25.6)	9.6 (6.2, 20.5)	0.659
Echocardiographic parameters			
LVEF (%)	63 (59, 66)	59 (57, 64)	0.164
LAVI (mml/m^2^)	40.70 (33.15, 54.00)	44.60 (31.80, 55.30)	0.656
TAPSE (cm)	2.60 (2.15, 2.95)	2.40 (2.20, 2.85)	0.532
E/A	1.10 (0.88, 1.40)	1.20 (0.92, 1.60)	0.478
E/e	8.25 (7.25, 10.45)	9.70 (8.30, 11.75)	**0.042**
Lateral E/e	7.30 (6.25, 9.55)	8.60 (6.90, 10.40)	0.126
Septal E/e	10.25 (8.77, 11.75)	11.50 (10.20, 14.10)	**0.032**
TR Vmax (cm/s)	242.00 (219.10, 271.55)	251.50 (216.85, 265.50)	0.937
Aortic Valve Stenosis			0.159
None (*n*, %)	38 (97.4)	33 (86.8)	
Mild (*n*, %)	0 (0.0)	3 (7.9)	
Moderate (*n*, %)	1 (2.6)	2 (5.3)	
Diastolic dysfunction (*n*, %)	7/28 (20.0/80.0)	9/28 (24.3/75.7)	0.875
HanDeCT (high/low risk) (*n*, %)	12/24 (33.3/66.7)	19/19 (50.0/50.0)	0.224

*Note:* Data are presented as median with IQR or numbers with percentages. Mann–Whitney‐*U*‐Test and Fisher's exact test have been used to calculate the *p*‐value. Values in bold indicate statistical significance (*p* < 0.05).

Abbreviations: ALD, Alcohol‐related Liver Disease; ALT, Alanine Aminotransferase; AP, Alkaline Phosphatase; AST, Aspartate Aminotransferase; CRP, C‐reactive protein; eGFR, estimated Glomerular Filtration Rate; FIPS, Freiburg Index of Post TIPS Survival; HanDeCT, Hannover algorithm to Detect the risk of Cardiac decompensation after TIPS; HE, Hepatic Encephalopathy; INR, International Normalised Ratio; LAVI, left atrial volume index; LVEF, left ventricular ejection fraction; MASLD, Metabolic Dysfunction‐Associated Steatotic Liver Disease; MELD, Model for End stage Liver Disease; MetALD, Metabolic and Alcohol‐associated Liver Disease; NT‐proBNP, N‐Terminal pro‐B‐type Natriuretic Peptide; PSE, Portosystemic Encephalopathy; TAPSE, Tricuspid Annular Plane Systolic Excursion; TIPS, Transjugular Intrahepatic Portosystemic Shunt; TR Vmax, Tricuspid Regurgitation Maximal Velocity; γGT, gamma Glutamyl Transferase.

To evaluate the clinical outcome in direct comparison to standard TIPS, a control cohort was generated using propensity score matching. 52 eligible cTIPS patients were matched 1:1 by Freiburg index of post TIPS survival (FIPS) against 49 patients who received sTIPS ≥ 8 mm between 10/2020 and 04/2023. The use of a caliper of 0.25 resulted in the allocation of 40 patients per group, thereby establishing the foundation for this manuscript (Figure 1B). Adverse events (death, paracentesis, variceal bleeding, HE, cardiac decompensation and TIPS‐thrombosis) were assessed for 12 months following TIPS insertion. TIPS‐thrombosis, as diagnosed by the treating physician, was confirmed using abdominal ultrasound or computed tomography [29]. In addition, TIPS‐revision during follow‐up was assessed.

Symptom control was defined as absence of variceal bleeding during follow‐up or need for paracentesis between months 4 and 12 after TIPS insertion, to account for the time needed for the portal pressure reduction to take full effect. If a full follow‐up of 365 days was not reached, symptom control was assessed at the end of follow‐up.

### 2.2 Placement of a Constrained TIPS With 6 mm Diameter

**FIGURE 2:**
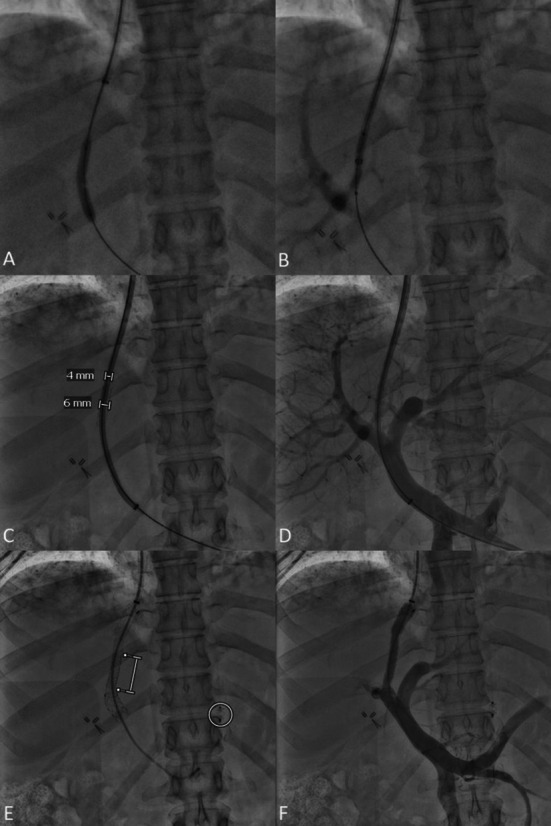
Placement of constrained TIPS with 6 mm diameter (A) After the right portal vein branch is successfully punctured, the intraparenchymal tract is dilated with a 6 mm balloon catheter (B) A 10‐French TIPS sheath is advanced and a 6 mm balloon‐expandable stent‐graft in positioned in the tract (C) The 6 mm balloon‐expandable stent‐graft is deployed and the TIPS sheath is gently passed through the stent‐graft in the portal vein branch. Of note, the outer diameter of the 10‐French TIPS sheath is 4 mm (D) A control portography is acquired (E) The TIPS endoprosthesis is positioned from the portal vein branch within the stent‐graft (bar) in the intraparenchymal tract to the hepatic vein and deployed. In addition, embolization of gastric varices can be performed to close and control collateral shunt flow and guide the blood flow through the TIPS (circle) (F) A control portography is conducted with a pigtail‐catheter in the portal vein to confirm sufficient flow through the constrained TIPS.

The standard pre‐interventional work‐up and TIPS procedure with image guidance according to established standard operating procedures were described elsewhere [30]. In standard procedure, TIPS were established under general anesthesia using a Siemens Artis Q or Siemens Artis Pheno angiography system (Siemens Healthcare, Germany). cTIPS were established by experienced interventional radiologists, who also performed the standard TIPS placements [30] (Figure 2). After ultrasound‐guided puncture of the right internal jugular vein, a 10‐French sheath was inserted and the TIPS needle (GORE TIPS Set, Gore, AZ, USA) was guided from the right hepatic vein into the right portal vein branch using fluoroscopy or image guidance (2D/3D overlay) [30]. An additional cone‐beam computed tomography was acquired in one patient and an additional transsplenic access was used in another patient to facilitate sTIPS placement [31]. In contrast to the sTIPS procedure, the puncture tract was dilated with a 6 mm balloon catheter (Mustang, Boston Scientific, MA, USA) and an available balloon‐expandable stent or stent‐graft of 5 mm to 7 mm in diameter (Gore Viabahn VBX Stent Graft, Gore, AZ, USA; Advanta V12 balloon‐expandable covered stent, GETINGE, Sweden; Omnilink Elite Vascular Balloon‐Expandable Stent, Abbott, IL, USA; Express LD, Boston Scientific, MA, USA) was placed in the intrahepatic tract through the 10F TIPS sheath. The diameter of this stent(−graft) was set to 6 mm. Thereafter, the TIPS endoprosthesis (Gore Viatorr with controlled expansion, Gore, AZ, USA) was advanced through the stent(−graft) and positioned to connect the right hepatic vein and the right portal vein branch. Thus, the diameter of the otherwise self‐expandable TIPS endoprosthesis was constrained to 6 mm by the surrounding stent(−graft). The TIPS endoprosthesis was then dilated with a 6 mm balloon catheter (Mustang, Boston Scientific, MA, USA) for proper alignment. Finally, a portography using a pigtail catheter in the portal vein was acquired with a standardised contrast injection protocol. The pressures in the inferior vena cava and in the portal vein were measured before and after TIPS placement and the portosystemic gradient (PSG) was calculated as recommended [32]. During the sTIPS procedure, the diameter of the TIPS endoprosthesis was initially set to 8 mm [30].

Additional variceal embolization (VE) was performed in patients with prominent gastroesophageal varices to close and control collateral shunt flow and guide the blood flow through the TIPS as recommended [32]. Post‐interventional monitoring was conducted at the intensive care unit or general ward. Of note, one patient in the sTIPS group received a TIPS endoprosthesis with a diameter of 8 mm under local anaesthesia through an occluded Wallstent 12/60 mm (WALLSTENT‐Uni, Boston Scientific, MA, USA) that was initially placed as TIPS with a 10 mm diameter 15 years ago.

### 2.3 Statistical Analysis

Statistical analysis was conducted using R 4.2.0 statistical computation system with R commander (http://www.jstatsoft.org/v14/i09/) and plugin EZR [33]. Continuous data are shown as median with interquartile range (IQR). Categorical data are presented as counts (percentages). Comparison of the data within cTIPS cohort and sTIPS cohort was conducted via two‐sided Wilcoxon test for related samples. Distinction of the data between cTIPS group and sTIPS group was achieved using two‐sided Mann–Whitney U test for continuous data or Chi squared test/Fisher's exact test for categorical data when appropriate.

Logistic regression was employed to measure the effect of cTIPS vs. sTIPS on symptom control and further decompensations. Incidence of adverse events after TIPS was analysed through Fine‐Grey proportional hazard competing risk analysis (competing event being death). Kaplan–Meier curves and Cox proportional hazard regression were used to depict survival. In all time‐dependent analyses, complete TIPS thrombosis without timely recanalization was considered a loss and censored at the time of diagnosis of the dysfunction. Level of significance was *p* < 0.05.

## 3 Results

### 3.1 Study Population

Patient demographics of cTIPS and sTIPS group, each composed of 40 patients, are summarised in Tables 1 and 2. Baseline characteristics were mostly not statistically different between the cTIPS and sTIPS group, showing appropriate matching of the two populations. As expected, cTIPS patients presented with lower cholinesterase, representative of decreased liver function (*p* = 0.005).

**TABLE 2:** Procedural characteristics of patients treated with either standard TIPS or constrained TIPS.

	Standard TIPS	Constrained TIPS	*p*‐value
PSG before (mmHg)	15 (13, 18)	15 (13, 17)	0.732
PSG after (mmHg)	6 (5, 8)	7 (6, 9)	**0.042**
Delta PSG (mmHg)	10 (7, 11)	7 (6, 9)	**0.025**
PSG reduction (%)	60 (53, 69)	50 (41, 58)	**0.006**
Dose Area Product (Gy*cm^2^)	108.82 (46.67, 199.51)	91.84 (52.27, 157.23)	0.780
Number of DSA series	4 (3, 6)	4 (3, 5)	0.087
Fluoroscopy Time (min)	15 (11, 23)	14 (10, 20)	0.630
Overall Procedure Time (min)	60 (44, 75)	51 (41, 64)	0.209
Variceal embolization (*n*, %)	25/15 (62.5/37.5)	27/13 (67.5/32.5)	0.815

*Note:* Data are presented as median with IQR or numbers with percentages. Mann–Whitney‐*U*‐Test and Fisher's exact test have been used to calculate the *p*‐value. Bold values indicate statistical significance (*p* < 0.05).

Abbreviations: DSA, digital subtraction angiography; PSG, portosystemic gradient; TIPS, Transjugular Intrahepatic Portosystemic Shunt; VE, Variceal embolization.

cTIPS patients were predominantly male (65%, *n* = 26), median age was 60.5 years, median model for end stage liver disease (MELD) score was 12, median FIPS −0.05. Most prevalent aetiology of liver cirrhosis was alcohol‐associated (75%, *n* = 30) and main indication for TIPS placement was refractory ascites (80%, *n* = 32). Within the cTIPS group indication for the individualised constrained approach was cardiac impairment (55%, *n* = 22), and/or DLF (43%, *n* = 17) and/or history of HE (20%, *n* = 8).

### 3.2 Technical Feasibility and Procedural Characteristics

The technical success rate for both cTIPS and sTIPS was 100%. PSG decreased significantly in the cTIPS group from 15 mmHg [13 mmHg; 17 mmHg] to 7 mmHg [6 mmHg; 9 mmHg] (*p* < 0.001), as well as in the sTIPS group from 15 mmHg [13 mmHg; 18 mmHg] to 6 mmHg [5 mmHg; 8 mmHg] (*p* < 0.001). PSG was not significantly different between cTIPS and sTIPS before TIPS (*p* = 0.732), but PSG remained significantly higher in the cTIPS cohort after TIPS placement (*p* = 0.042). In accordance, PSG Delta (10 mmHg [7 mmHg; 11 mmHg] in sTIPS vs. 7 mmHg [6 mmHg; 9 mmHg] in cTIPS) and percentage PSG reduction (60% [53%; 69%] in sTIPS vs. 50% [41%; 58%] in cTIPS) were significantly lower in cTIPS patients (*p* = 0.025 and *p* = 0.006, respectively). Details are given in Table 3.

**TABLE 3:** Univariate logistic regression for the influence of treatment with standard versus constrained TIPS on symptom control during 1 year after TIPS.

	Standard TIPS	Constrained TIPS	OR	95% CI	*p‐value*
Variceal bleeding (*n*, %)	7(17.5)	5 (12.5)	0.67	0.19–2.33	0.533
Paracentesis (*n*, %)	16 (40.0)	20 (50.0)	1.50	0.62–3.64	0.370
Paracentesis between months 4 and 12 after TIPS (*n*, %)	5 (12.5)	10 (25.0)	2.33	0.72–7.59	0.159
Lack of Symptom control (*n*, %)	9 (22.5)	15 (37.5)	2.07	0.78–5.51	0.147

Abbreviations: CI, Confidence Interval; OR, Odds ratio; TIPS, Transjugular Intrahepatic Portosystemic Shunt.

The overall procedural time of 51 min [41; 64] of the cTIPS group was not statistically different to 60 min [44; 75] of the sTIPS group (*p* = 0.209). Fluoroscopy time was comparable in both groups, with 15 min [10; 20] in the cTIPS group and 15 min [11; 23] in the sTIPS group (*p* = 0.630). The number of digital subtraction angiography (DSA) series was not statistically different between both groups (cTIPS 4 [3; 5] vs. sTIPS 4 [3; 6]; *p* = 0.087). Furthermore, the dose area product (DAP) of 91.84 Gy*cm^2^ [52.27; 157.23] in the group with cTIPS was relatively low compared to the DAP of 108.82 Gy*cm^2^ [46.67Gy*cm^2^; 199.51Gy*cm^2^] in the sTIPS group, but there was no statistical significance (*p* = 0.780). The number of patients, who received VE during the TIPS placement, was not different between the cTIPS group (67.5%, *n* = 27) and sTIPS group (62.5%, *n* = 25) (*p* = 0.815).

### 3.3 Clinical Outcome and Adverse Events

In sTIPS patients, symptoms were insufficiently controlled in 22.5% (*n* = 9), while 15 patients (37.5%) presented with bleeding or persistent need for paracentesis after cTIPS. However, this lack of symptom control did not differ significantly between the two groups (*p* = 0.147; OR 2.07 [0.78–5.51] for cTIPS) (Table 3).

**FIGURE 3:**
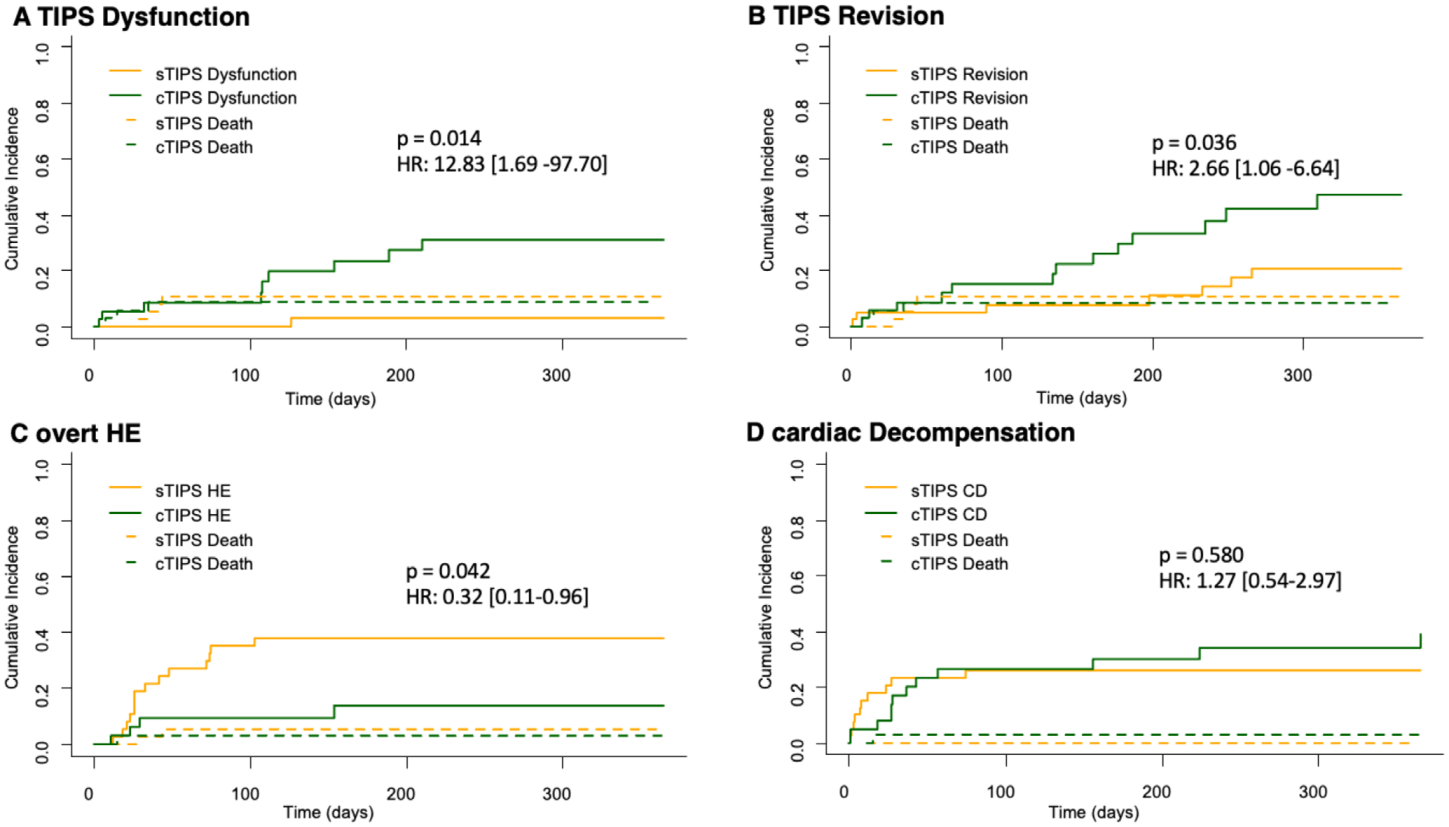
Influence of treatment with standard versus constrained TIPS on clinical outcomes and adverse events during 1 year after TIPS in competing risk analysis (Competitor: Death). (A) Influence of treatment with sTIPS versus cTIPS on the incidence of TIPS dysfunction (B) Influence of treatment with sTIPS versus cTIPS on the incidence of TIPS revision (C) Influence of treatment with sTIPS versus cTIPS on the incidence of overt hepatic encephalopathy (D) Influence of treatment with sTIPS versus cTIPS on the incidence of cardiac decompensation.

In contrast, TIPS dysfunction due to thrombosis was significantly more frequent in the cTIPS cohort (*n* = 9 (22.5%)) than in sTIPS (*n* = 1 (2.5%)) (*p* = 0.014; HR 12.83 [1.69–97.70]) in competing risk analysis (Table 4, Figure 3A). Importantly, in case of thrombosis, the TIPS can be revised by balloon dilatation to recanalize the lumen (8 recanalization interventions, 7 of which were successful, in cTIPS; no recanalization attempt in sTIPS). Moreover, in the sense of this individualized step‐up approach, balloon dilatation was used to increase the diameter in patients with insufficient symptom control (*n* = 5 in cTIPS, *n* = 5 in sTIPS). While no cTIPS had to be reduced or occluded due to clinical complications, intervention had to take place in 2 sTIPS patients (Table 4). In one patient, the TIPS diameter had to be decreased because of recurring HE and CD. Another sTIPS patient developed acute liver failure shortly after TIPS, so that the sTIPS had to be transiently occluded, but was reopened once stabilised. Overall, the need for revision (balloon dilatation in case of thrombosis and/or insufficient symptom control, reduction or occlusion) remained higher in the cTIPS group (*n* = 7 (17.5%) in sTIPS vs. *n* = 13 (32.5%) in cTIPS, *p* = 0.036; HR 2.66 [1.06–6.64]) (Table 4, Figure 3A).

**TABLE 4:** TIPS dysfunction and need for interventional TIPS revision during 1 year after TIPS.

	Standard TIPS	Constrained TIPS
**TIPS thrombosis (*n*, %)**	1 (2.5)	9 (22.5)
Recanalization after thrombosis (*n*, %)	0 (0.0)	7 successful (17.5) 1 unsuccessful (2.5)
Dilatation in case of lacking symptom control (*n*, %)	5 (12.5)	5 (12.5)
Reduction in case of complication (*n*, %)	2 (5.0)	0 (0.0)
**TIPS revision interventions in total (*n*, %)**	7 (17.5)	13 (32.5)

Abbreviation: TIPS, Transjugular Intrahepatic Portosystemic Shunt.

**FIGURE 4:**
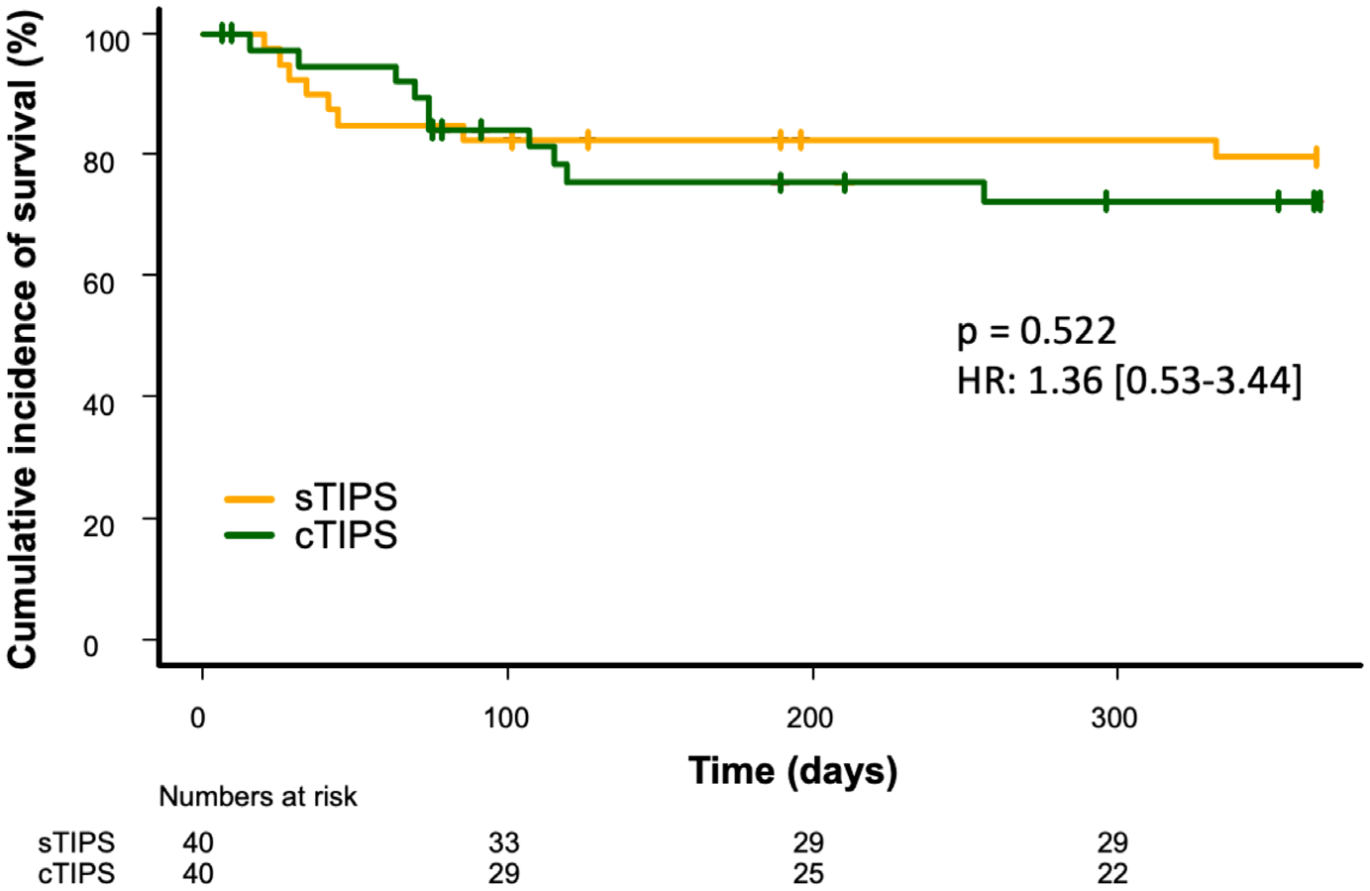
1 year survival after TIPS insertion comparing patients treated with constrained and standard TIPS.

Patients treated with cTIPS developed significantly less overt HE during follow‐up (*n* = 14 (35%) vs. 4 (10%); *p* = 0.042; HR 0.32 [0.11–0.96]) (Figure 3C), while there was no significant difference concerning the development of cardiac decompensation (*n* = 10 (25%) sTIPS vs. 12 (30%) cTIPS; *p* = 0.580; HR [0.54–2.97]) (Figure 3D). Over the course of the 12‐month follow‐up period after TIPS insertion 8 patients of the sTIPS group and 10 patients of the cTIPS group died, leaving a similar overall survival of 80% and 75%, respectively (*p* = 0.522; HR 1.36 [0.53–3.44]) (Figure 4).

## 4 Discussion

Optimal titration of the TIPS flow and control of shunt volume are key to preserving patients at risk from HE, CD or DLF. A conventional TIPS prosthesis with an 8 mm diameter has a similar shunt function compared to a 10 mm diameter, but reduces the risk of HE and hepatic dysfunction [5]. In a meta‐analysis, overall survival of patients is greater with covered stents of 8 mm diameter than with 10 mm diameter although the shunt dysfunction rate is opposite [34]. Cardiovascular and neurological co‐morbidities are frequent in patients with advanced liver cirrhosis, especially considering the aging population. Even in patients with relative contraindications, the risk of over‐shunting exists with an 8 mm TIPS. Therefore, there might be an increasing need for smaller TIPS diameters. Schepis et al. have evaluated results concerning post‐TIPS HE when performing tract under‐dilatation of conventional (8 or 10 mm) TIPS prostheses to only 6 mm in 42 patients [6]. While the results were promising with regard to the development of HE, the group has reported passive expansion of the TIPS prosthesis over time, which is supported by a study by Pieper et al. [6, 35]. Thus, passive expansion of the TIPS prosthesis renders the final diameter of the stent‐graft. Therefore, a true calibration of the PSG cannot be achieved through underdilated TIPS [6, 20, 35]. In patients with over‐shunting, various reduction techniques are described [8, 9, 10, 11, 12, 13, 17, 18, 19]. In consideration of complex TIPS reductions during later follow‐up, it seems favorable to primarily constrain a TIPS to a 6 mm diameter. If needed, the TIPS diameter can then be adjusted with simple balloon dilatation based on the clinical course of the patient in follow‐up examinations. This facilitates a controlled, stepwise reduction of the portosystemic gradient to the optimal value adapted to the patients' symptoms. It might be beneficial for patients with relative contraindications, increase the number of patients eligible for TIPS and improve survival and quality of life in these patients.

Reduction of the TIPS prosthesis during the initial placement has been reported using a constrained technique with a balloon expandable stent, which has shown stable diameter in the short term [15, 24, 25]. In the study by Cui et al., a patient cohort using constrained TIPS of 6 mm (*n* = 14), 7 mm (*n* = 5) and 8 mm (*n* = 9) new onset of HE has been reported, but no correlation with the TIPS diameter has been found [25]. In contrast, Rabei et al. have reported on 6 mm TIPS (*n* = 9) in comparison to conventional TIPS (*n* = 18) and HE has only been observed in the conventional group [24]. In accordance, Yan et al. published a significantly increased incidence of overt HE in 27.6% in an 8 mm TIPS group (*n* = 58) compared to 12.1% in a 6 mm TIPS group (*n* = 58) [15].

Our study presents the largest European cTIPS cohort to date. We found patients treated with cTIPS in our center to also develop significantly less overt HE. Of note, the frequency of TIPS dysfunctions due to thrombosis was increased in the cTIPS group when compared to the sTIPS group. This has also been described by Cui et al. [25]. Most likely, this is due to the decreased total shunt volume, which facilitates clotting and stasis. Nevertheless, the reported TIPS dysfunction rate of 22.5% during 12 months in the cTIPS group is in line with early TIPS thrombosis rates of 5% to 25% found in the literature [12, 36, 37]. Importantly, the survival of cTIPS patients was not impacted.

In contrast to prior studies [24, 25], we used a controlled expansion TIPS prosthesis in our study. As reported by Praktiknjo et al., the diameter of this TIPS prosthesis is stable at 8 mm without passive expansion to 10 mm [21]. Therefore, this TIPS prosthesis might be favorable for a constrained approach due to limited expansion force and a lower risk of passive expansion. Although the additional placement of a balloon expandable stent(−graft) increases costs, an adjustable diameter might broaden the indication for TIPS in patients with relative contraindications. Moreover, expensive TIPS reductions in decompensated patients can be avoided.

Pre‐interventional PSG of both groups was not different. Percentage PSG reduction was significantly lower in the cTIPS group with 50%, compared to the sTIPS group with 60%. This reflects the expected hemodynamic effect on the portal system with a TIPS of 6 mm diameter instead of 8 mm diameter [5, 6, 25] and is favorable to preserve portal flow to the liver and limit the otherwise excessive cardiac preload after TIPS insertion. Nevertheless, the post‐TIPS PSG of 7 mmHg in our cohort was in line with the post‐TIPS PSG of 6.1 mmHg in the cTIPS group published by Rabei et al. [24] and deemed the intervention successful, as a PSG below 12 mmHg is a recommendation for conventional TIPS placement [3, 28, 32].

Additional VE have been performed in both groups. In the cTIPS group, VE reduces the blood flow in competitive portosystemic collateral pathways and increases the blood flow through the TIPS tract. This might be a key point in 6 mm TIPS, because VE thereby acts as a protective measure against TIPS thrombosis. Overall, the radiation exposure and procedure time of the cTIPS group are not statistically different compared to the sTIPS group. This indicates no prolongation of the procedure by additional VE and/or additional stent(−graft) placement.

However, there are several study limitations. We assessed an individualised approach to TIPS, expanding accessibility for otherwise ineligible patients. Therefore, this study has a retrospective approach and is not prospectively randomised due to ethical reasons. This newly introduced TIPS technique was only performed at a single tertiary care and transplantation center, limiting the patient number. In addition, the longer‐term patency and expansion behaviour of the diameter of the cTIPS prosthesis need to be assessed in follow‐up examinations. Since this approach was designed to aid a clinical gap, rather than generate prospective study data, there was no interventional surveillance of the cTIPS prosthesis, but rather standard clinical follow‐up. Therefore, prospective, randomised multi‐center studies with extended follow‐up and additional matching for cardiac function are required to further generate evidence for the use of the cTIPS technique. Given the complex interaction between TIPS diameter, portovenous collateral flow and anticoagulation, study design will be challenging. Moreover, the effect of general anaesthesia, implemented in the Baveno VII consensus statement [32], and also the impact of concomitant variceal embolization on PSG‐measurements are further limitations. Measurement and interpretation of absolute PSG values and percentage changes during the intervention and in follow‐up examinations will be another important point for patient selection which needs further investigation. Finally, whether the placement of an additional stent(−graft) in the beginning and balloon‐dilatation in follow‐up examinations outweigh the costs of TIPS reductions or improve cost‐effectiveness by reduced morbidity and mortality of high‐risk patients cannot be answered at the moment and needs economic evaluation of long‐term data.

In conclusion, cTIPS with an endoprosthesis in stent(−graft) placement is feasible and safe. It facilitates size‐adjustable TIPS placement in patients requiring small‐diameter TIPS prostheses due to increased risk for post‐interventional HE, CD or DLF and has the potential to broaden the spectrum of patients who are eligible for TIPS.

## Author Contributions

All authors contributed either to research design (H.R., T.C.M., B.M. and J.B.H.) and/or the acquisition (H.R., T.C.M., J.B.H, A.T., and J.B.M.) analysis (H.R.) or interpretation (all authors) of data. H.R. and T.C.M. drafted the manuscript, which was critically revised by all other authors.

## Funding

This work was supported by Else Kröner‐Fresenius‐Stiftung. PRACTIS Clinician Scientist Program, DFG ME (3696/3).

## Ethics Statement

This study was approved by the local ethics committee of Hannover Medical School (Nr. 7935_BO_K_2018) and followed the principles of the Declaration of Helsinki. All patients provided written informed consent. The Hannover TIPS patient registry is registered at clinicaltrials.gov as NCT04801290.

## Conflicts of Interest

B.M. reports lecture and/or consultant fees from AbbVie, Fujirebio, Gilead, Luvos, MSD, Norgine, Roche, W. L. Gore & Associates. He received research support from Altona, EWIMED, Fujirebio and Roche. H.W. reports lecture and/or consultant fees from Abbott, Bristol‐Myers‐Squibb, Hoffmann‐La Roche, Roche, Gilead, GlaxoSmithKline, Janssen, Vir Biotechnology. He also received research support from Abbott and Biotest. F.K.W. reports institutional research support from Delcath Systems, Siemens Healthineers, Philips Healthcare, Promedicus Ltd., Becton, Dickinson and Company. All other authors disclose no potential financial or non‐financial conflicts of interest regarding this study.

## Data Availability

The data that support the findings of this study are available on request from the corresponding author. The data are not publicly available due to privacy or ethical restrictions.
